# Pore Size Independent Particle Size Control of Mesoporous N‐doped Carbon Nanospheres for 3D Bottom‐Up Electrode Design

**DOI:** 10.1002/smll.202506253

**Published:** 2025-11-29

**Authors:** Niklas Ortlieb, María B. Camarada, Olivia Basu, Hafiz M. N. Amin, S. Esmael Balaghi, Anna Fischer

**Affiliations:** ^1^ Institute of Inorganic and Analytic Chemistry University of Freiburg Albertstraße 21 79104 Freiburg Germany; ^2^ Freiburg Center of Interactive Materials and Bioinspired Technologies (FIT) University of Freiburg Georges‐Köhler‐Allee 105 79110 Freiburg Germany; ^3^ Cluster of Excellence livMatS University of Freiburg Georges‐Köhler‐Allee 105 79110 Freiburg Germany; ^4^ Freiburg Materials Research Center (FMF) University of Freiburg Stefan‐Meier‐Str. 21 79104 Freiburg Germany

**Keywords:** 3D bottom‐up electrode design, electrochemical energy conversion and storage, mesoporous N‐doped carbon nanospheres, particle size control, supercapacitors

## Abstract

The rational design of electrodes is crucial for improving electrochemical energy storage and conversion devices. High‐performance devices require porous carbon electrodes with controlled intraparticle properties — such as morphology, size, porosity, elemental composition, and graphitic microstructure — and interparticle features like electrode‐level porosity, percolation pathways, and tortuosity, that influence mass transport. Here, mesoporous N‐doped carbon (MPNC) nanospheres with independently tunable particle size at a fixed pore size is reported. Extending the previously established synthesis toolbox, independent control over particle and pore sizes is demonstrated. Using a 9 nm SiO_2_ hard template, particle sizes between 50 and 300 nm is adjusted while maintaining comparable physicochemical properties. These MPNC nanospheres are evaluated as supercapacitor electrodes in coin cells using 1.0 m LiPF_6_ in EC/DEC as electrolyte. The highest specific capacitance — 67 F g^−1^ at 0.1 A g^−1^ — is obtained with the largest particles, attributed to reduced tortuosity and improved electrode percolation. As all samples exhibited similar surface areas (≈950 m^2^ g^−1^), performance differences highlight particle size‐dependent diffusion limitations. This study establishes a bottom‐up approach for engineering electrode architectures, enabling independent control of pore and particle sizes of MPNC nanospheres and providing a platform to systematically investigate their effects on electrochemical performance.

## Graphical abstract

This work extends the previously published carbon toolbox for particle size independent pore size control of mesoporous N‐doped carbon nanospheres, with pore size independent particle size control, which, for the first time, allows the fully independent pore and particle size control of such carbon nanospheres and enables the true bottom‐up electrode design for electrochemical energy conversion and storage applications.

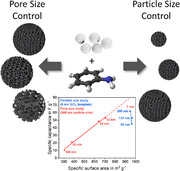

## 1 Introduction

The global shift toward renewable energy sources demands the development of advanced electrochemical energy conversion and storage devices such as fuel cells, electrolyzers, supercapacitors, and batteries. In such applications, carbon materials play a crucial role due to their structural versatility and electrode design possibilities, serving as conductive supports for high‐performance electrocatalysts, active storage materials in supercapacitors, and active material, structural component, or conductive additives in battery electrodes.^[^ ^1^, ^2^, ^3^, ^4^, ^5^ ^]^

The electrochemical performance of such energy devices depends significantly on the rational design of the carbon electrode structure. Efficient electrodes require well‐defined 3D intraparticle porous architectures within the carbon particle volume as well as interparticle porosity within the whole electrode volume. Intraparticle porosity, encompassing pore size, connectivity, pore volume, and surface area, directly influences ion accessibility and charge storage capabilities. Simultaneously, interparticle porosity governs the packing density, percolation, and tortuosity of the electrode, which affect electrolyte distribution, mass transport, and overall conductivity.^[^ ^6^, ^7^, ^8^ ^]^

**Figure 1:**
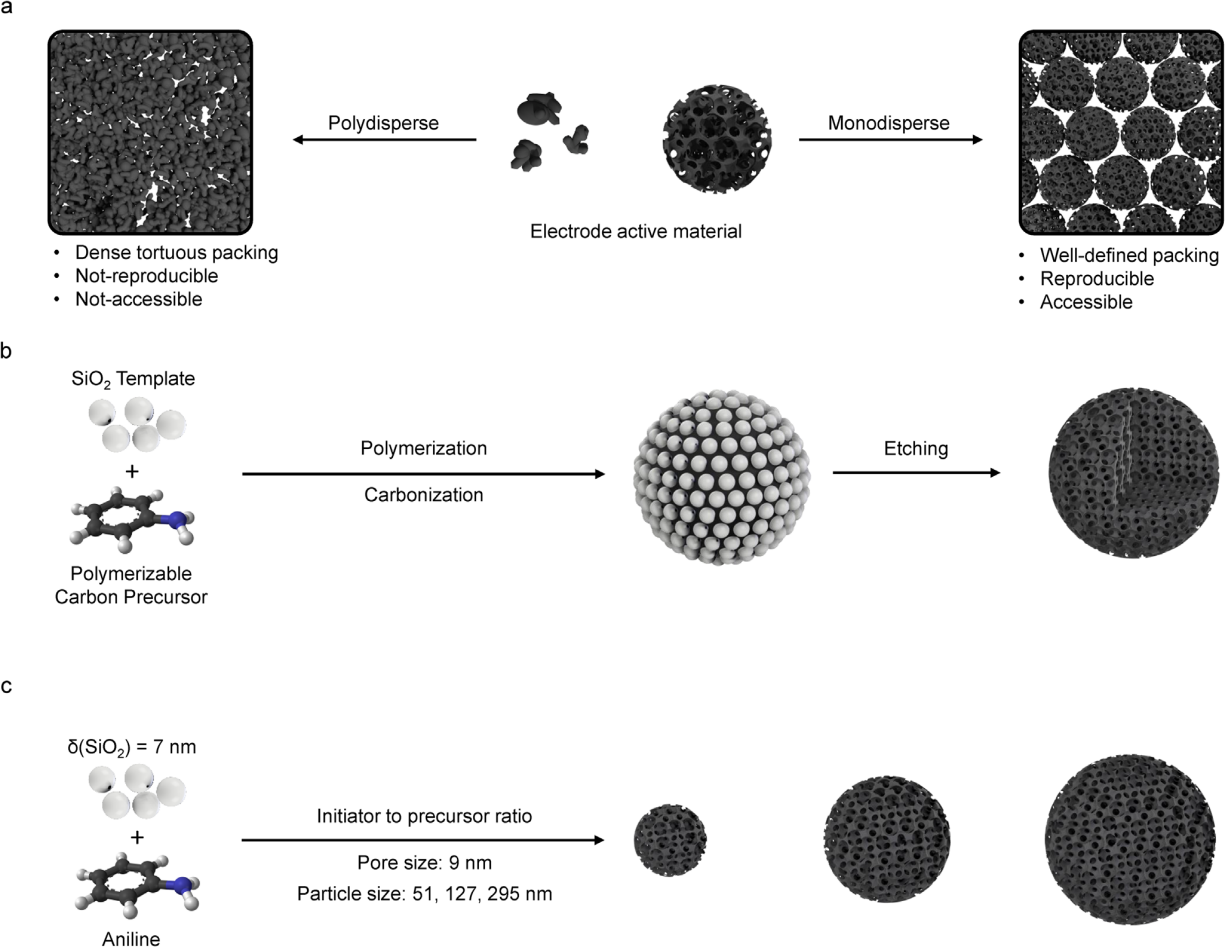
Influence of the particle size and particle size distribution on the structure of processed electrodes (a). While polydisperse commercial carbon particles (left) form dense and tortuous structures, monodisperse MPNC nanospheres (right) form homogeneously percolated electrodes with interparticle pores defined by the particle size. Synthesis scheme with the general approach (b) and particle size engineering by changing the molar initiator to carbon precursor ratio (c). The particle size can be changed without affecting the pore size. Adapted with permission from Wiley.^[^ ^5^ ^]^

Typically, carbon electrodes are assembled from carbon powders, worked up with solvents, binders, and other additives into inks, slurries, or pastes, which are then homogeneously coated onto selected current collectors. The dried mixture forms a 3D percolated electrode with a structure defined by the primary particle morphology and size, as well as secondary agglomerates. Therefore, the primary particle properties have a crucial impact on the electrode structure and the resulting electrochemical performance of the device. Polydisperse samples with undefined particle shapes and sizes lead to random particle packing in the electrode structure, which affects coating reproducibility. This also results in a higher tortuosity of the pore channels, which leads to a worse percolation of the electrode and, ultimately, to a non‐accessible “dead” internal electrode volume (**Figure** **1**a). In contrast, monodisperse spherical particles form homogeneous and defined electrode structures with interparticle pores defined by the particle size of the carbon spheres.^[^ ^9^, ^10^, ^11^ ^]^

Mesoporous carbons are well‐suited as electrode material for the mentioned applications due to their large specific surface area, good conductivity, and chemical stability.^[^ ^12^ ^]^ Nitrogen doping of the carbon structure can additionally increase the conductivity of the material and tune the polarity of the surface, which is crucial for the dispersion of the carbon particles in the slurry or paste used for electrode production.^[^ ^13^, ^14^ ^]^ Nevertheless, control over the intraparticle properties like pore and particle size, chemical and surface composition, or degree of graphitization is necessary to allow the efficient 3D bottom‐up electrode design for electrochemical applications.

The two main approaches for the synthesis of mesoporous carbon nanoparticles are based on soft‐ and hard‐templating strategies, which can utilize a large variety of carbon precursors and nanometer‐sized templates.^[^ ^15^, ^16^, ^17^, ^18^, ^19^, ^20^, ^21^, ^22^, ^23^ ^]^ After fixing the carbon structure during carbonization and template removal, (meso)porous carbon nanomaterials can be obtained in which the pores reflect the size and shape of the used template. While soft‐templating is based on structure‐directing agents, such as surfactants or block‐copolymers, that self‐assemble in micelles which serve as templates, hard‐templating is based on the polymerization or thermal condensation of a carbon precursor around a thermally stable inorganic template such as metal, metal salt, or metal oxide nanoparticles.^[^ ^24^, ^25^, ^26^, ^27^, ^28^, ^29^, ^30^, ^31^ ^]^ In the literature, hard‐templating has proven to be a reliable approach with excellent robustness, reproducibility, and scalability due to the variability of templates and their good thermal stability, which allows for precise control over porous structures in carbon materials. One such hard‐templating approach is the synthesis of self‐assembled polyaniline‐silica composite nanoparticles, which can be converted into porous carbon nanoparticles by carbonization and template removal.

Aniline‐based mesoporous N‐doped carbon (MPNC) nanospheres were first described by Wang et al.^[^ ^19^ ^]^ They used silica nanospheres as a hard template and ammonium persulfate to initiate the polymerization of aniline into polyaniline, resulting in polyaniline‐silica composite materials with the template particles homogeneously distributed within the polyaniline matrix. After carbonization and etching, carbon nanospheres with sizes of 98, 222, and 264 nm were obtained. However, the increase in particle size was only possible when the template size was increased (from 7 to 42 nm), and thus, no pore size‐independent particle size control was achieved. Similarly, Nan and Yue reported the SiO_2_ hard‐templated synthesis of MPNC nanospheres with diameters of 300–500 nm. However, the particle size was again linked to the size of the used SiO_2_ template and could not be adjusted independently.^[^ ^21^ ^]^

In one of our previous studies published by Melke et al., in contrast, we were able to change the particle size by diluting the reaction solution using the same approach.^[^ ^20^ ^]^ This resulted in particle sizes of 470–170 nm for a given pore size of 24 nm, depending on the dilution. The authors attributed this finding to the change of the pH, but it remained unclear whether it was actually the pH or the dilution of all reactants that led to the decrease in particle size, as the molar ratios of the reactants to each other remained constant by the dilution. Additionally, when the template size was changed from 12 to 41 nm, the particle size changed from 180 to 320 nm, as previously described by Wang et al.^[^ ^19^ ^]^

These examples clearly show that despite certain progress in the particle size control of MPNC nanospheres, no pore size‐independent particle size control has been achieved yet. This, however, would be necessary to enable rational bottom‐up electrode design.

In our previous study, we were able to synthesize MPNC nanospheres with five different pore sizes between 16 and 99 nm without changing their particle size of around 300 nm.^[^ ^5^ ^]^ The synthesis was based on a silica hard‐templating approach, and material characterization showed that besides the surface area and the pore volume, all other investigated physicochemical parameters (particle morphology and size, degree of graphitization, and elemental composition) were kept constant for all materials, and thus, the only changed material parameter was the pore size. The synthesized materials were then used as electrode materials for supercapacitors as a model application to demonstrate pore size effects independently from any particle size effect. With decreasing pore size and thus increasing surface area, the capacitance of the electrode increased, and a linear correlation between the MPNC surface area and the resulting specific capacitance was obtained. The best performance was achieved for MPNC‐12‐300‐1000 based electrodes (with 12 indicating the template diameter in nm, 300 the particle size in nm, and 1000 the carbonization temperature in °C), resulting in gravimetric capacitances of 55 F g^−1^ at 0.5 A g^−1^ in two‐electrode coin cells using 1.0 m LiPF_6_ in EC/DEC as electrolyte. These values were slightly lower than comparable systems from the literature, but our systems were characterized by high capacitance retentions of over 80% even at high charge/discharge rates of 10 A g^−1^, which outperformed many literature reports and could be attributed to the homogeneously percolated electrode structure.^[^ ^32^, ^33^, ^34^, ^35^, ^36^, ^37^ ^]^

Here, we report a follow‐up material engineering study, where we varied the particle size between 51 and 295 nm for a given pore size of 9 nm (template size 7 nm) using the same synthetic approach (Figure 1b) but using varying molar initiator to carbon precursor ratios of 1:10, 1:2, and 1:1 (Figure 1c). The MPNC nanospheres were obtained with narrow particle size distributions (PDI < 1.15), a controlled degree of graphitization governed by the carbonization temperature and nitrogen content imprinted by the aniline carbon precursor. The obtained nanospheres had surface areas in the range of 967–945 m^2^ g^−1^ and pore volumes of 1.903–1.213 cm^3^ g^−1^. We again used the synthesized MPNC nanospheres as electrode material for supercapacitors as a model application to investigate the influence of the interparticle porosity of the electrode on the performance of the system. This resulted in specific capacitances between 48 and 67 F g^−1^ at 0.1 A g^−1^ in a two‐electrode coin cell system, again using 1.0 m LiPF_6_ in EC/DEC as electrolyte. The specific capacitance hereby decreased with decreasing particle size, which could be attributed to particle size‐dependent diffusion limitations within the percolated electrode structure, where denser particle packings increase the tortuosity of the electrode and thereby decrease the capacitance.

With these results, the present study represents a second axis of our MPNC toolbox, now allowing the synthesis of MPNC nanospheres within a wide range of independently adjustable pore and particle sizes. At the same time, all other investigated physicochemical parameters of the synthesized carbon materials (particle morphology, degree of graphitization, and elemental composition) remain constant. This not only enables researchers to study pore and particle size effects independently from each other in model electrodes but also opens the doors to a tailored bottom‐up electrode design for several electrochemical applications for energy storage and conversion technologies, and beyond.

## 2 Results and Discussion

### 2.1 Material Synthesis and Characterization

The mesoporous N‐doped carbon (MPNC) nanosphere synthesis strategy was based on the oxidative polymerization of aniline in the presence of silica (SiO_2_) nanoparticles, as previously reported by us.^[^ ^2^, ^5^, ^20^, ^38^, ^39^, ^40^, ^41^, ^42^ ^]^ Through an electrostatically enhanced self‐assembly process, polyaniline (PANI) chains and SiO_2_ nanoparticles co‐assembled into monodisperse spherical composite particles, in which the SiO_2_ nanoparticles were uniformly embedded within a PANI matrix. Hereby, the initiator ammonium persulfate (APS) was consumed stoichiometrically and thus played a crucial role in the chain length of the PANI, the self‐assembly process, and the particle size of the resulting composite materials.^[^ ^19^, ^43^, ^44^ ^]^ Carbonization and template removal by etching with ammonium hydrogen difluoride (NH_4_(HF_2_)) then resulted in MPNC nanospheres with accessible mesopores.

Based on the mechanism proposed by Wang et al. and our understanding of the self‐assembly process, both SiO_2_ and PANI are positively charged under the reaction conditions and repel each other.^[^ ^19^ ^]^ At the same time, sulfate anions are formed during the polymerization, which can act as an electrostatic “glue” to bind SiO_2_ and polyaniline chains together. As soon as a critical sulfate concentration is reached, an electrostatically enhanced self‐assembly process occurs, leading to spherical composite particles. A lower initiator concentration has two effects, resulting in smaller particles. First, the probability of two PANI chains becoming entangled is lower when the chain length is shorter. Second, a lower sulfate concentration means a reduced probability of self‐assembly.

**Figure 2:**
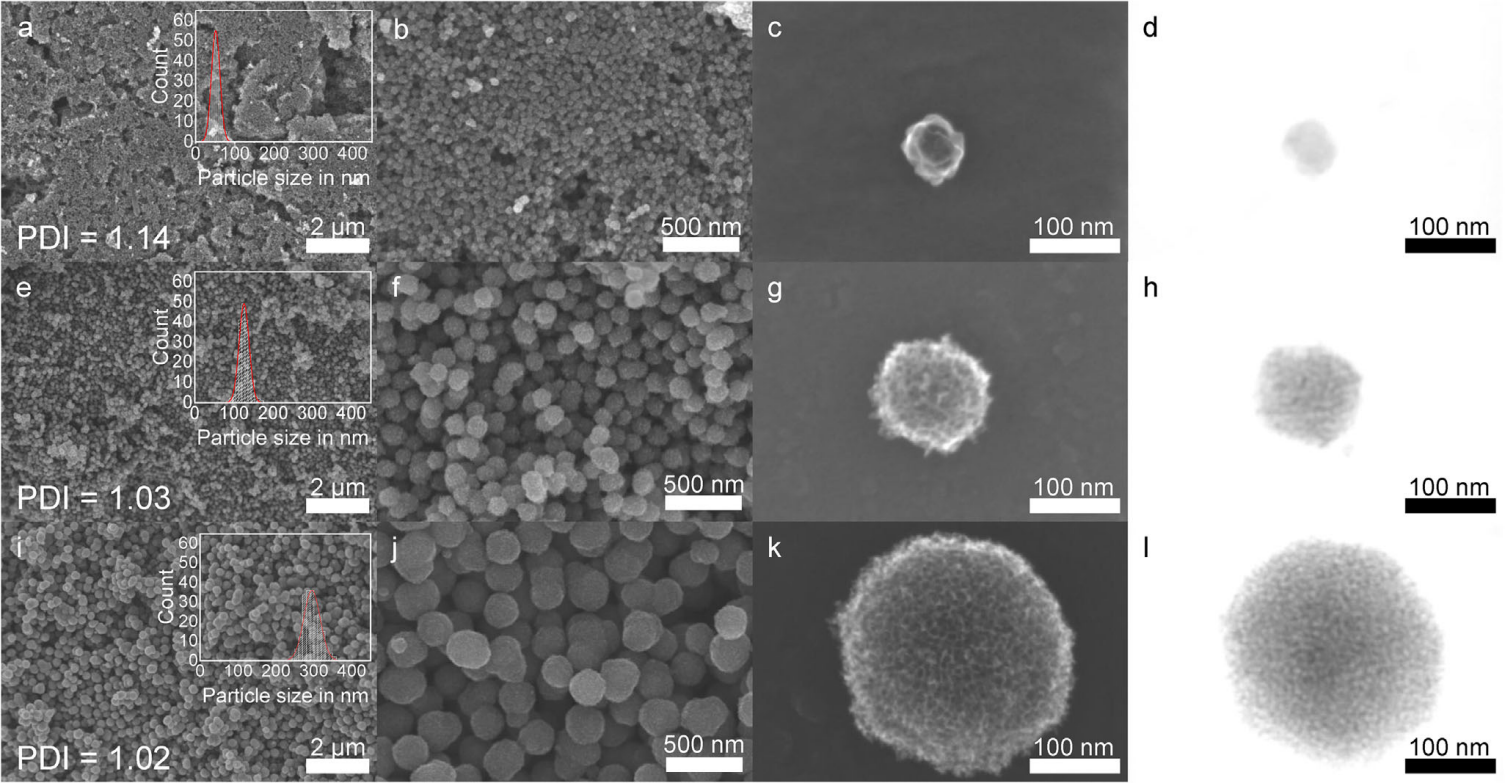
SEM and STEM images of the synthesized MPNC nanospheres with different particle sizes for MPNC‐7‐50‐1000 (a–d), MPNC‐7‐130‐1000 (e–h), and MPNC‐7‐300‐1000 (i‐l). Insets show the particle size distributions of the synthesized MPNC nanospheres. A polydispersity (PDI) Đ < 1.1 is defined as monodisperse according to the IUPAC definition.^[^ ^45^ ^]^

Using different molar APS to aniline ratios (1:10, 1:2 and 1:1) while keeping all other synthesis parameters constant, we tailored the MPNC nanosphere particle size in the range from 50 to 300 nm (51 ± 11, 127 ± 13, 295 ± 22 nm) as can be seen in the scanning (transmission) electron microscopy (SEM/STEM) images in **Figure** **2** (a larger version of the particle size distributions can be found in Figure S1, Supporting Information). The samples were denoted as MPNC‐TS‐PS‐PT with TS = template size, PS = particle size, and PT = pyrolysis temperature. It should be noted that the resulting pore sizes are slightly larger than the nominal template size, due to the synthesis process. However, we stuck to the nominal template size for the sample names. As discussed above, the particle size distribution of the MPNC nanospheres plays an important role in the electrode structure formed after processing and thus should ideally be narrow and, in the best case, monodisperse (Polydispersity Index (PDI) Ð <1.1 regarding IUPAC definition).^[^ ^45^ ^]^ This is the case for all three synthesized samples. However, it is noteworthy that the PDI of the samples increased with decreasing particle size, but is still within the definition of monodispersity regarding IUPAC, with values of 1.02 for MPNC‐7‐300‐1000 and 1.03 for MPNC‐7‐130‐1000. Only MPNC‐7‐50‐1000 showed a value of 1.14, which is slightly above the limit defined for a monodisperse sample. Nevertheless, the absolute standard deviation for MPNC‐7‐50‐1000, with a value of 11 nm, shows that the particle size distribution of the sample is still narrow. The calculated increased PDI arose from the very small synthesized particle size, where already slight variations of the particle size can lead to large changes in the PDI. Besides the particle size distribution, all MPNC samples showed a homogeneous pore distribution within the particle volume with highly accessible pores, as shown by STEM images (Figure 2d,h,l). Additional HRTEM images can be found in the supporting information (Figure S2, Supporting Information).

**Figure 3:**
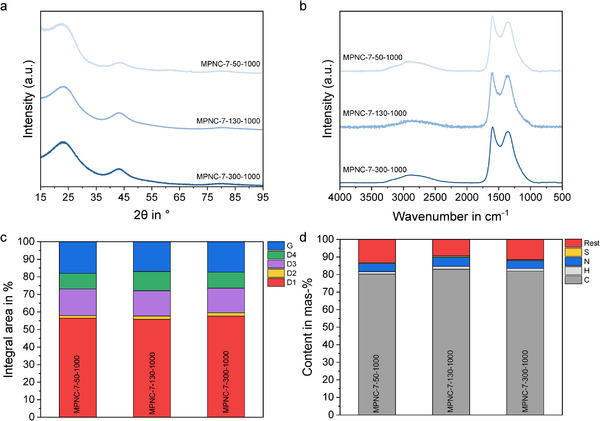
XRD patterns (a) and Raman spectra (b), normalized integrated areas of the fitted Raman bands for all samples using a five‐band (G, D1, D2, D3, D4) model with different types of microstructural spectroscopic contributions (c) and elemental combustion analysis (d) for the MPNC nanospheres with different particle sizes.

The microstructure of the MPNC nanospheres with different particle sizes was evaluated using X‐ray diffraction (XRD) and Raman spectroscopy (**Figure** **3**a,b). XRD analysis showed comparable diffractograms for all samples, with reflections at 2θ = 23.5° and 43.7° (λ = 1.54 Å, Cu Kα) assigned to the (002) and (100) reflections characteristic of disordered carbon materials.^[^ ^46^ ^]^ Raman analysis of the MPNC nanospheres demonstrated similar microstructures of the synthesized materials, independently of the particle size, too. The two visible Raman bands resulting from defect‐free (G‐band) and defect‐rich (D1‐band, edges of graphene sheets, point defects, dopants, polyaromatic clusters) at 1605 and 1365 cm^−1^, were fitted with a five‐band model, deconvoluting the two main bands with additional three defect bands at 1670 (D2, top layers of graphite stacks), 1533 (D3, amorphous carbon) and 1160 cm‐1 (D4, polyenes or sp^2^ hybridized carbon atoms bound to sp^3^ hybridized carbon atoms).^[^ ^47^, ^48^ ^]^ The deconvolution (Figure S3, Supporting Information) led to a degree of graphitization (defined by the ratio of the area of the G‐band to the overall area of the spectrum) of 17.4 ± 0.5%. The defect bands were in the range of 56.7 ± 0.9%, 1.7± 0.2%, 14.5± 0.5% and 10.0± 1.0% for D1, D2, D3 and D4, respectively (Figure 3c; Table S1, Supporting Information). These results are in line with our first study on MPNC nanospheres, where we adjusted the pore size for a set particle size of 300 nm.^[^ ^5^ ^]^ In both cases, the samples were carbonized at 1000 °C, which resulted in a comparable carbon microstructure. Elemental combustion analysis (Figure 3d; Table S2, Supporting Information) resulted in values of 81.6 ± 1.4, 1.5 ± 0.1, 5.0 ± 0.3, and 0.6 ± 0.2wt.%, for carbon, hydrogen, nitrogen, and sulfur, showing similar elemental compositions for all three samples and are again comparable with our previous study.

**Figure 4:**
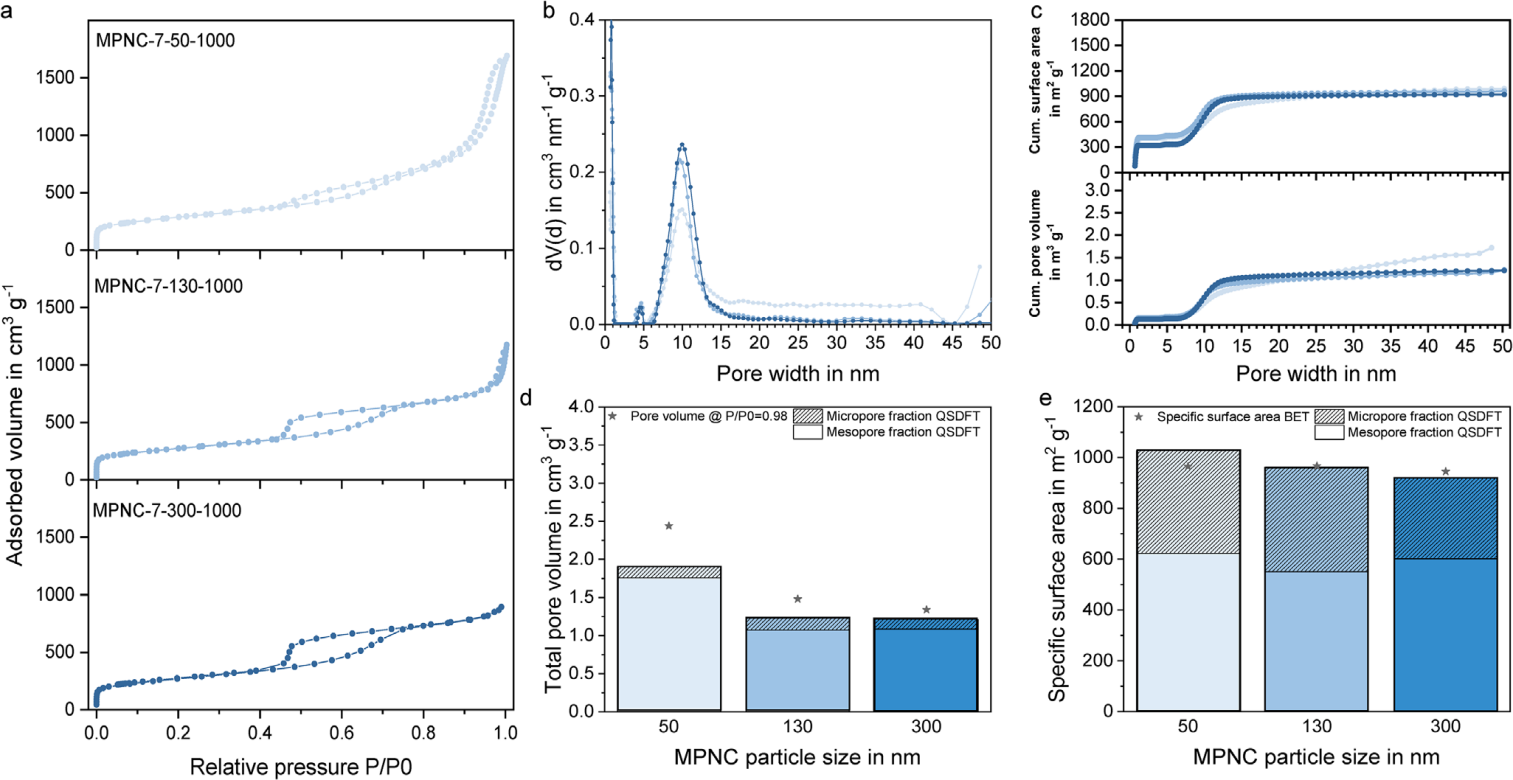
Nitrogen physisorption isotherms (a), pore size distribution (b), cumulative pore volume and surface area (c), the corresponding total pore volume (d), and specific surface area (e) of the MPNC samples with different particle sizes.

Finally, the pore structures of the MPNC nanospheres with different particle sizes were evaluated by nitrogen physisorption measurements. For all three samples, type IV isotherms with pronounced hysteresis loops were obtained, indicating connected and accessible mesopores in the nanospheres (**Figure** **4**a).^[^ ^49^ ^]^ The pore size distribution determined by QSDFT (Figure 4b) revealed micropores in the materials and a main mesopore size of 9 nm, which is slightly bigger than the nominal SiO_2_ nanoparticle template size of the used template (7 nm). While the difference between nominal template size and resulting pore size in the MPNC nanospheres seems to be large at first sight, particle size analysis of the template with SEM and short angle X‐ray scattering (SAXS) demonstrated an average particle size between 8.5 and 9.5 nm (Figure S4, Supporting Information), even if a nominal particle size of 7 nm was defined by the manufacturer of the SiO_2_. Thus, the average pore size determined by QSDFT is well within the range of the used template size. Of course, the fitting of the theoretical isotherms to the experimentally determined isotherm in the QSDFT calculations could lead to a slightly incorrect pore size determination, as well as swelling of the template during synthesis or structural shrinking of the carbon during carbonization or etching. However, we consider these effects to be negligible. The cumulative pore volume and surface area (Figure 4c) followed the expected trend with significantly increasing values around the average pore diameter of 9 nm. With decreasing particle size, the MPNC nanospheres showed a slight increase in the pore volume and the specific surface area (Figure 4d,e). While the difference was negligible between MPNC‐7‐300‐1000 and MPNC‐7‐130‐1000, the increase was significant between MPNC‐7‐130‐1000 and MPNC‐7‐50‐1000, especially for the pore volume. This effect can be explained by the formation of interparticle pores between the packed nanoparticles in the measurement cell. With decreasing particle size, the interparticle pore size decreases. For very small particles, the interparticle pore size is then in the mesopore range, leading to an increased measured pore volume. For the surface area, the effect of the interparticle pores is much smaller, as the internal surface area of the nanospheres is much higher compared to the external surface area. In the series of MPNC nanospheres in the particle size study, it is clearly visible, that this was the case for MPNC‐7‐50‐1000, resulting in a specific surface area of 965 m^2^ g^−1^ and a pore volume of 2.439 cm^3^ g^−1^, while MPNC‐7‐130‐1000 and MPNC‐7‐300‐1000 showed values of 967 and 945 m^2^ g^−1^ as well as 1.481 and 1.341 cm^3^ g^−1^, respectively (Table S3, Supporting Information). Hereby, the results clearly demonstrated that the intraparticle surface area of the MPNC nanospheres had to be much larger than their outer surface area, as a decrease of the nanosphere diameter from 300 to 50 nm usually would result in an increase of the outer surface area by a factor of 36. When comparing the micropore and mesopore fractions of the samples, it becomes evident, that this trend was in fact an effect of the formed interparticle pores, as the micropore fraction resulting from the internal pores or surface of the particles was nearly constant for all three samples, showing values of 367 ± 47 m^2^ g^−1^ and 0.142 ± 0.015 cm^3^ g^−1^. Only the values of MPNC‐7‐300‐1000 were slightly smaller.

### 2.2 Electrochemical Characterization

As in our previous pore size study, we used electrochemical double‐layer capacitor (EDLC) measurements as a model application to study particle size effects at constant intraparticulate pore size on the EDLC performance due to the sensitivity of the application to changes in specific surface area and mass transport limitations. For this purpose, we produced MPNC nanosphere containing inks using polyvinylidene difluoride (PVDF) as a binder and doctor‐bladed electrodes on aluminium foil as a current collector. Circular electrodes with a diameter of 12 mm were then used in symmetrical EDLCs in CR2032 coin cell housings with a Whatman paper as separator and 1.0 m LiPF_6_ in EC/DEC as electrolyte. The electrode loading was in the range of 0.7 mg cm^−2^ for all electrodes. It is noteworthy that exactly the same experimental setup, including the electrode size and loading, separator, and electrolyte, was used to allow a direct and meaningful comparison to our previously published study, as changes in these parameters could massively influence the electrode performance.

**Figure 5:**
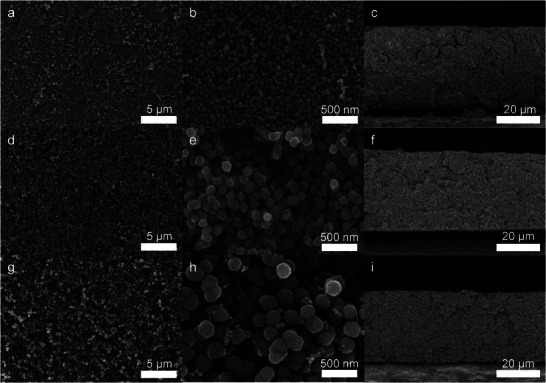
SEM images and cross‐sections of the coatings prepared from the MPNC‐based inks: MPNC‐7‐50‐1000 (a–c), MPNC‐7‐130‐1000 (d–f), and MPNC‐7‐300‐1000 (g–i). Insets show zoomed‐in areas of the cross‐sections.

SEM images of the coated electrodes can be seen in **Figure** **5** and show homogeneous electrode structures for all three MPNC nanosphere sizes. In the top views, the different particle sizes and their packing can be clearly seen. From the images, it becomes evident that the interparticle porosity decreases with decreasing particle size as discussed above. The cross‐sections revealed a homogeneous percolation not only on the top surface of the electrodes but within the whole electrode volume. Nevertheless, it is clearly visible that the smaller MPNC nanospheres formed a denser‐packed, more tortuous electrode structure compared to the larger nanospheres. Finally, the thickness of the electrodes measured from the cross‐sections resulted in similar values of 35 ±3 µm for all electrodes, with only a small decrease from 38 to 32 µm with increasing particle size.

**Figure 6:**
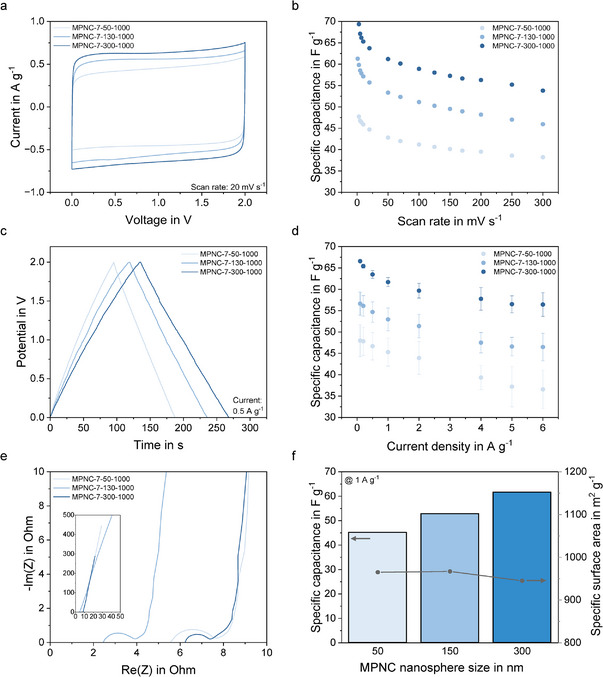
Evaluation of the performance of the MPNC‐based EDLCs in coin cell housings using 1.0 m LiPF6 in EC/DEC as electrolyte. Comparison of cyclic voltammograms recorded at 20 mV s^−1^ for all MPNC nanospheres with different particle sizes (a) and specific capacitance dependent on the scan rate (b). Galvanostatic charge‐discharge curves obtained at 0.5 A g^−1^ (c) and correlation between the applied current and specific capacitance calculated from the GCD profiles (d). Nyquist plots of the MPNC‐based EDLC devices (e) and overview of the specific capacitance of the MPNC Nanospheres with different particle sizes compared to the specific surface area.

Cyclic voltammetry (CV) of the coin cells was performed in the range of 0–2 V (**Figure** **6**a). CVs of all samples recorded with scan rates from 1 to 300 mV s^−1^ can be found in Figure S5 (Supporting Information). Independent of the used MPNC nanosphere size, quasi‐rectangular CV shapes with large areas under the curve were obtained, indicating superior EDLC performance of all electrodes. Even at very low scan rates of 1 mV s^−1^ or high scan rates of 300 mV s^−1^ the quasi‐rectangular shape was maintained, correlating with only a very small electrochemical polarization resistance during the charge/discharge process. Calculated specific capacitances for MPNC‐7‐50‐1000 had values of 47.7 F g^−1^ at a scan rate of 1 mV s^−1^ and 38.2 F g^−1^ at 300 mV s^−1^, showing a scan rate‐dependent capacitance decrease with a capacitance retention of 80% (Figure 6b). For MPNC‐7‐130‐1000, these values increased to 61.3 F g^−1^ at a scan rate of 1 mV s^−1^ and 45.9 F g^−1^ at 300 mV s^−1^, with a capacitance retention of 75%. For MPNC‐7‐300‐1000 values of 69.4 F g^−1^ at a scan rate of 1 mV s^−1^, 53.8 F g^−1^ at 300 mV s^−1^ and a capacity retention of 78% were measured, respectively. While a decrease in the specific capacitance with increasing scan rate was expected due to limited ion diffusion from the electrolyte into the pores of the MPNC nanospheres at higher scan rates, the obtained capacitance retentions were extraordinarily high for the use of mesoporous carbon materials and the given electrode thickness.

The same trends were obtained in galvanostatic charge‐discharge (GCD) experiments (Figure 6c; Figure S6, Supporting Information). Here, ideal EDLC behaviour is indicated by a symmetrical triangular shape of the charge‐discharge curve with no features at the top or bottom vertices between charge and discharge direction, which would indicate voltage drops due to ohmic losses. Again, all three samples followed the expected triangular trend, indicating their good performance as EDLC. As for the CV measurements, the lowest specific capacitance was obtained for the MPNC‐7‐50‐1000 based electrode with values of 48 F g^−1^ at a charge/discharge current of 0.1 A g^−1^ and 37 F g^−1^ at a charge/discharge current of 6 A g^−1^, translating into a capacitance retention of 77%. With increasing MPNC nanosphere size, the obtained capacitances increased as was the case in the CV measurement, resulting in initial specific capacitances of 57 and 67 F g^−1^ at a charge/discharge current of 0.1 A g^−1^ for MPNC‐7‐130‐1000 and MPNC‐7‐300‐1000‐based electrodes, respectively. At high charge/discharge currents of 6.0 A g^−1^ values of 47 and 56 F g^−1^ were measured for these two electrode types, resulting in high capacitance retentions of 83 and 84%.

To understand the particle size effects of the MPNC nanospheres on the EDLC performance, the obtained capacitances need to be linked with the structure of the electrodes. As described above, the interparticle pore size depends on the particle size of the MPNC nanospheres and thus, smaller nanospheres lead to smaller interparticle pore sizes. This results in a denser and more tortuous electrode structure, which limits the ion diffusion through the percolated electrode structure and could possibly lead to inaccessible “dead” electrode volume. At the same time, the intraparticle porosity of the three MPNC nanosphere samples is similar, as it was demonstrated by the physisorption measurements. This also explains the great rate capability of all electrodes, which can be attributed to the intraparticle porous structure of the MPNC nanospheres. The interconnected mesopores provide good electrolyte infiltration and efficient ion diffusion within the intraparticle porosity of the nanospheres, allowing for high charge/discharge currents. However, with increasing particle size, the intraparticle tortuosity of the MPNC nanospheres increases, which could also lead to ion diffusion limitations. When comparing the specific capacitances of the three MPNC nanosphere‐based electrodes with different particle sizes, it becomes evident that the values are decreasing with decreasing particle size and thus, denser electrode packings, which concludes that the effect of the electrode percolation is larger than the effect of the intraparticle tortuosity (at least for 9 nm mesopores). Additionally, the electrode thickness slightly increased with decreasing particle size, possibly amplifying the tortuosity effect of the electrode structure.

These thoughts are underlined by the impedance measurements in the frequency region of 10 mHz to 300 kHz, measured at the open circuit potential of the coin cells (Figure 6e). The Nyquist plots showed only small ohmic contributions from the coin cells (i.e., intrinsic resistance of the MPNC nanospheres, the bulk resistance of the electrolyte, and the contact resistance of the electrode and the current collector),^[^ ^50^ ^]^ represented by the small equivalent series resistances (ESR) visible as the intercepts of the spectra with the real axis of the plot. Starting from the intersection, a semicircle is formed for all three electrodes, indicating the charge transfer resistance of the electrodes.^[^ ^51^ ^]^ Interestingly, the diameter of the semicircle and thus the charge transfer resistance increases with decreasing particle size, with values of 1.2, 1.6, and 2.3 Ω for the MPNC‐7‐300‐1000, MPNC‐7‐130‐1000, and MPNC‐7‐50‐1000 based electrode, respectively. This correlates well with the interparticle porosity of the electrodes, with denser and more tortuous electrode structures for decreasing nanosphere sizes. In the middle frequency region, a Warburg element represented by a linear section of the spectra with an angle of around 45° is visible. In an EDLC, this element is crucial because it indicates the resistance of the ion diffusion within the pores of the active material.^[^ ^50^ ^]^ It becomes clearly visible that the length of the linear section increased with increased nanosphere size, indicating diffusion limitations with increasing tortuosity of the intraparticle porosity. Nevertheless, the length of all Warburg elements is relatively small, showing good diffusive behavior of all three electrodes. Finally, a nearly vertical line is visible in the spectra in the low frequency region, correlating with the EDLC charge storage, e.g., the capacitive behavior of the electric double layer formed at the electrode/electrolyte interface.^[^ ^52^ ^]^ Here, for a given frequency, a lower endpoint of the spectra indicates a higher capacitance of the electrode. The lowest endpoint was found for the MPNC‐7‐300‐1000 based electrode, which is consistent with the results of the CV and GCD measurements.

The particle size effect on the EDLC performance is also summarized in Figure 6f. From the graph, it becomes evident that the specific capacitance of the MPNC nanospheres with different particle sizes is not correlated with the specific surface area, as was the case in our previous pore size study, but decreases with decreasing particle size and thus a denser, more tortuous electrode structure. The best EDLC performance was hereby achieved by the MPNC‐7‐300‐1000 based electrode with a high specific capacitance of 67 F g^−1^ at a charge/discharge current of 0.1 A g^−1^ and a superior capacity retention of 84% at a charge/discharge current of 6 A g^−1^.

**Figure 7:**
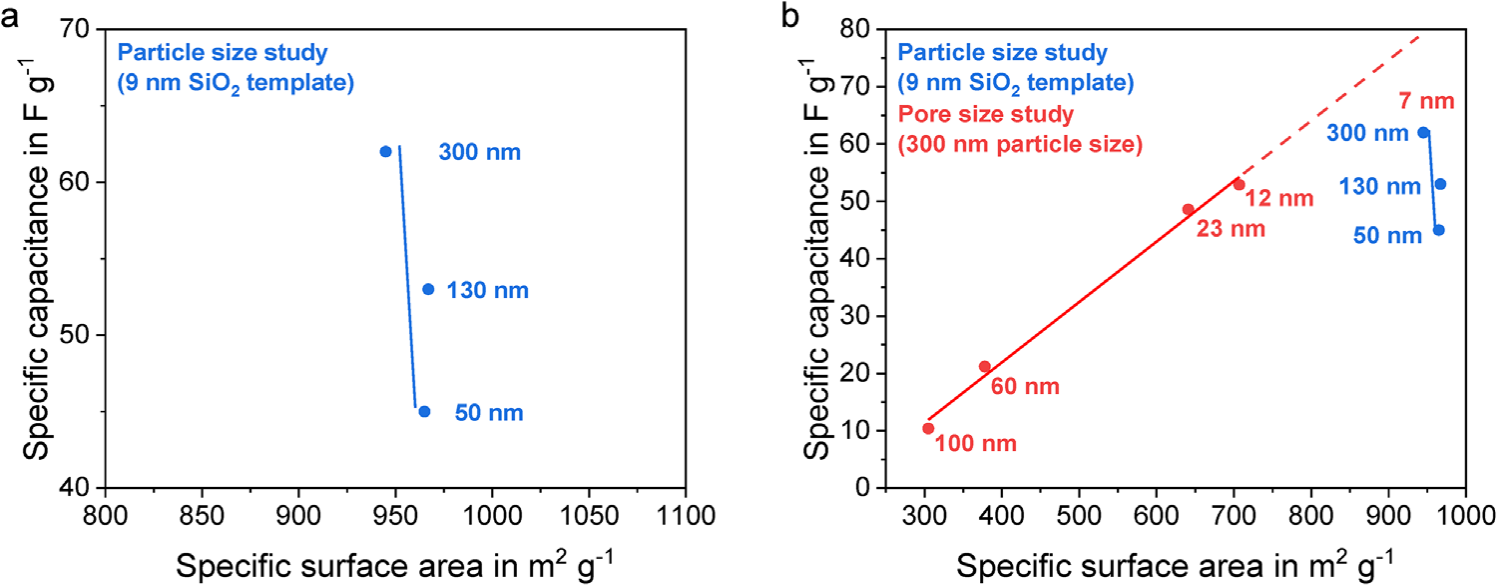
Particle size dependent specific capacitance and specific surface area of MPNC nanosphere based electrodes in this work (a). Comparison of the specific capacitance results from the MPNC nanosphere particle size study in this work with our previous results from the MPNC nanosphere pore size study (b). All specific capacitance values are given for a charge/discharge current of 1 A g^−1^. Adapted with permission of Wiley.^[^ ^5^ ^]^

Plotting the specific capacitance of the investigated electrodes against the specific surface area of the used MPNC nanospheres (**Figure** **7**a) visualizes this effect even better, clearly showing the reduced specific capacitance with decreasing particle size, while the specific surface area remained nearly constant for all three particle sizes. A comparison with the EDLC results from our previous MPNC nanosphere pore size study is shown in Figure 7b.^[^ ^5^ ^]^ While for the previous pore size study, the specific surface area and thus the specific capacitance increased with decreasing pore size and a linear correlation was obtained between the specific capacitance and specific surface area, the current particle size study clearly demonstrated, that decreasing particle sizes lead to a denser and more tortuous electrode structure, limiting the maximal achievable specific capacitance for a given pore size and thus specific surface area. Overall, the MPNC‐7‐300‐1000‐based electrode still clearly outperformed the best MPNC‐12‐300‐1000‐based electrode from the pore size study, as the specific surface area was further increased. However, the previously obtained linear trend could not be maintained with the increased specific surface area. This could mean that the described intraparticulate tortuosity reached its limit and thus the interparticle diffusion limitation started to play a role at this small pore size, or that the electrode was too thick to have electrolyte percolation within the whole electrode volume. Further studies with even smaller mesopore sizes could clearly demonstrate if this is the case.

Compared to the literature, the maximal obtained specific capacitance of 64 F g^−1^ at a charge/discharge current of 0.5 A g^−1^ for the MPNC‐7‐300‐1000 based coin cell is still lower than some of the reported systems but already in the range of other reports. For example, Liu et al. synthesized mesoporous carbon nanoparticles with 4.6 nm pore size and 450 to 500 nm particle size, that had a surface area of 788 m^2^ g^−1^. They obtained a specific capacitance of 190 F g^−1^ at a charge/discharge current of 0.5 A g^−1^ using 6 m KOH as electrolyte, while their capacity retention was only 69% at 8 A g^−1^.^[^ ^36^ ^]^ In contrast, Zhang et al. synthesized mesoporous carbon particles with a pore size of 3.5 nm and a particle size of 630 nm, showing a higher surface area of 1009 m^2^ g^−1^. While their surface area was larger compared to the carbon materials in our work, they only reached a specific capacitance of 55.8 F g^−1^ at a charge discharge current of 0.5 A g^−1^ and a capacitance retention of 70% at 10 A g^−1^ using again 6 m KOH as electrolyte, and thus being outperformed by our system.^[^ ^37^ ^]^ Organic electrolytes with wider electrochemical stability windows have also been reported.^[^ ^53^ ^]^ For example, Zou et al. used 1 m tetraethylammonium tetrafluoroborate (TEABF_4_) in propylene carbonate (PC) with highly mesoporous carbon flakes derived from kapok fibers (BET surface area after KOH activation ≈3010 m^2^ g^−1^), achieving a specific capacitance of ≈140 F g^−1^ at 0.5 A g^−1^.^[^ ^54^ ^]^

However, comparisons across studies are inherently limited by differences in electrolytes, carbon precursors, and testing protocols. The aim of this work was not to demonstrate a record‐performance supercapacitor, but to disentangle the effects of pore and particle sizes of MPNC nanospheres on EDLC behavior. Targeted optimization of the MPNC nanospheres could further improve performance: chemical or physical activation (e.g., KOH, CO_2_/steam) may increase accessible surface area by introducing additional microporosity, thereby boosting gravimetric capacitance, while preserving mesopores should sustain fast mass transport and high‐rate capability. With appropriate control of pore size distribution, heteroatom content, and particle morphology, MPNC‐based electrodes could approach commercially relevant capacitances at practical mass loadings and current densities.

## 3 Conclusion

This work introduced the pore size‐independent particle size control of monodisperse mesoporous N‐doped carbon (MPNC) nanospheres as a second axis in our previously reported MPNC toolbox. This advancement enables the synthesis of MPNC nanospheres with independently adjustable pore and particle sizes, unlocking precise, tailored 3D bottom‐up electrode design for electrochemical energy storage and conversion applications, and beyond.

The particle size adjustment was achieved by controlling the molar initiator to aniline ratio (1:10, 1:2, and 1:1), resulting in monodisperse particle sizes of 51 ± 11, 127 ± 13, and 295 ± 22 nm for a given SiO_2_ hard‐templated pore size of 9 nm (template size of 7 nm). While the particle size was adjusted, all other investigated physicochemical parameters were kept constant, resulting in degrees of graphitization in the range of 17.4 ± 0.5% and a nitrogen content of 5.0 ± 0.3 wt.%. At the same time, a high specific surface area around 956 ± 11 m^2^ g^−1^ was achieved and maintained constant independently of the particle size. The specific pore volume, in turn, increased with decreasing particle size, as the interparticle pores decreased into the mesopore range for the MPNC‐7‐50‐1000 sample and thus could be determined by nitrogen physisorption, while larger interparticle macropores between the nanospheres of the MPNC‐7‐130‐1000 and MPNC‐7‐300‐1000 samples could not be measured with this method.

The synthesized samples were then tested as electrodes in symmetrical coin cells for their performance as electrochemical double‐layer capacitors (EDLCs), due to the sensitivity to the specific surface area and ion diffusion limitations of this application. Galvanostatic charge‐discharge measurements resulted in increasing specific capacitances with increasing particle size, showing values of 48, 57, and 67 F g^−1^ at 0.1 A g^−1^ for MPNC‐7‐50‐1000, MPNC‐7‐130‐1000, and MPNC‐7‐300‐1000 based electrodes. At the same time, the electrodes showed superior rate capability due to their controlled and homogeneous (meso)porous structure, resulting in capacity retentions of 77, 83, and 84%, even at high charge/discharge currents of 6 A g^−1^.

In conclusion, we were able to link the performance of the electrodes with particle size effects and showed that smaller particles lead to a denser and more tortuous electrode structure that limits the specific capacitance by ion diffusion. In comparison to our previous pore size study, where we could linearly link the specific capacitance with the specific surface area of the MPNC nanospheres, we were able to further increase the specific capacitance in this work for the MPNC‐7‐300‐1000‐based electrode by using a smaller pore size and thus having a higher specific surface. However, the use of the smaller pore size led to a loss of the linear correlation, possibly due to a starting ion diffusion limitation within the intraparticle porosity of the MPNC nanospheres.

## 4 Experimental Section

### Materials/Chemicals

Silica (SiO_2_) nanoparticles with a nominal size of 7 nm, hydrochloric acid (HCl, 1.0 m), ammonium persulfate (APS, ≥98.0%), and ammonium hydrogen difluoride (NH_4_HF_2_, ≥95%) were purchased from Sigma–Aldrich. Aniline (99.5%) was purchased from VWR. Lithium hexafluorophosphate (1.0 m) in ethylene carbonate/diethyl carbonate electrolyte (1.0 m LiPF_6_ EC/DEC = 50/50) was purchased from Elyte. Carbon‐coated Al foil (≥99.9%) was purchased from Cam‐energy.

### Synthesis of Mesoporous N‐doped Carbon Nanospheres

Mesoporous N‐doped carbon (MPNC) nanospheres were synthesized via an oxidative polymerization of aniline in the presence of SiO_2_ nanoparticles with a size of 7 nm as a hard template, as previously reported.^[^ ^5^ ^]^ In a 4000 mL flask, 3200 mL MilliQ H_2_O, 400 mL 1.0 m HCl, 32.0 mL aniline, and the volume of SiO_2_ hard template solution containing 38.4 g SiO_2_ were added subsequently under stirring, while the flask was cooled in an ice bath. While the homogeneous dispersion cooled down, 80.20 g ammonium persulfate (APS) was dissolved in 160 mL of 1.00 m HCl. The APS solution was added dropwise to the reaction solution over 15 min, using a dropping funnel, and the mixture was stirred for 24 h while keeping the reaction flask cooled in the ice bath. The resulting polyaniline‐silica‐composite material was collected by centrifugation and washed until the pH of the supernatant was neutral. The washed composite material was then subjected to freeze‐drying to prevent any morphological changes while drying and subsequently carbonized under N_2_ for 6 h at 1000 °C (heating ramp: 200 K h^−1^). The SiO_2_ template was removed by etching with 4.0 m NH_4_(HF_2_) solution (50 mL g^−1^ composite) for 48 h. The MPNC nanospheres were separated from the etching solution by filtration and washed with neutral water and ethanol. After trying under vacuum, the samples were heat treated under N_2_ at 500 °C for 2 h (heating ramp: 200 K h^−1^), to remove insoluble residuals from etching. The final MPNC nanosphere powder was obtained with a yield of 12.1 g and was denoted as MPNC‐7‐300‐1000 according to the template size, particle size, and carbonization temperature. MPNC‐7‐130‐1000 and MPNC‐7‐50‐1000 were synthesized using only 50% and 10% APS, resulting in yields of 6 and 1.2 g, respectively.

### Material Characterization

Scanning Electron Microscopy (SEM) images were obtained using a Field Emission Gun High‐Resolution SEM (FEG‐HRSEM) *SU8220* from Hitachi equipped with a SE (secondary electron), BSE (back‐scattered electron), and TE (transmission electron) detector. Images were measured in scanning mode at an accelerating voltage of 2.0 kV and a working distance of 3.0 mm. Scanning Transmission Electron Microscopy (STEM) images were measured on the same system at an acceleration voltage of 30 kV and a working distance of 8.0 mm using the transmission mode. The polydispersity index (PDI) of the MPNC nanospheres was calculated according to the IUPAC definition using Equation (1):^[^ ^45^ ^]^

(1) $$PDI=\frac{\sum N_{i}\sum N_{i}d_{i}^{4}}{\sum N_{i}d_{i}\sum N_{i}d_{i}^{3}}$$

where N_i_ denotes the number of particles with the diameter d_i_.

X‐ray diffraction was measured in Bragg‐Brentano configuration on a Bruker D8 Discover using a flat sample holder with an embedded silicon monocrystal. Diffractograms were recorded between 10° and 105° 2Θ with a step size of 0.025° and a measurement time of 0.5 s per step, while the sample was rotated at 15 rpm.

Raman measurements were obtained using a Senterra II microscope from Bruker equipped with a 532 nm laser. The laser power was set to 0.25 mW, and the wavenumber resolution was 4 cm^−1^. Spectra were recorded in the range of 750–2500 cm^−1^ with 500 coadditions and a measurement time of 10 s per spectrum. Fitting of the spectra between 1000 and 1800 cm^−1^ was done with the Origin Lab 2024 software using a five‐band model.

A vario MICRO cube from Elementar Analysensysteme GmbH with a temperature programmable column and a thermal conductivity detector was used for elemental combustion analysis. Roughly one milligram of the sample was mixed with the same amount of WO_3_ as an oxidation catalyst and combusted at 1150 °C.

Nitrogen Physisorption was measured on a Micro300 physisorption station from 3P Instruments at 77 K. Prior to the measurements, all samples were degassed under vacuum at 200 °C for 6 h. Specific surface areas were calculated using the Brunauer–Emmer–Teller (BET) method, fitting the P/P_0_ range from 0.05 to 0.30. Pore size distributions were determined according to quenched solid density functional theory (QSDFT), considering slit, cylindrical, and spherical pores. The micropore‐specific surface areas and pore volumes were estimated using the *t*‐plot method and QSDFT equilibrium model.

### Supercapacitor Measurements

For the supercapacitor measurements, a CR2032 stainless steel coin cell housing was used. Two MPNC‐electrodes (12 mm diameter) separated by an electrolyte soaked (1.0 m LiPF_6_ in EC/DEC) Whatman paper (14 mm diameter) were used as the supercapacitor electrodes.

MPNC electrodes were prepared by dissolution of 10 mg of polyvinylidene difluoride (PVDF) binder in 1 mL of N‐methyl‐2‐pyrrolidone (NMP) via magnetic stirring for at least 12 h, followed by the addition of 80 mg MPNC powder and 10 mg of Super C65 as conductive additive to the clear solution. The obtained mixture was homogenized five times in a planetary dispenser (Kakuhunter SK‐300SII) for at least 2 min at 2000 rpm. The as‐prepared ink was then doctor‐bladed onto a carbon‐coated aluminium foil using a ZUA2000 blade and an MSK_AFA‐II Automatic Thick Film Coater with the thickness set to 250 µm at a speed of 10 mm s^−1^. The film was first dried for 1 h on a hot plate at 80 °C and then dried overnight in a vacuum oven at 50 °C, yielding an electrode loading of roughly 0.7 mg cm^−2^.

Cyclic voltammetry (CV) and galvanostatic charge‐discharge (GCD) measurements were performed in the voltage range of 0–2 V. Electrochemical impedance spectroscopy (EIS) was performed in the frequency range of 300 kHz to 10 mHz at the open circuit voltage with an alternate current amplitude of 5 mA. For all coin cell devices, triplicates were measured.

### Data Analysis for Electrochemical Double Layer Capacitance Performance

The specific capacitance C_s_ (in F g^−1^) of the electrodes based on the CV measurements was calculated with Equation (2), including the integral area, the mass m of the active material on the electrode, the potential or voltage window ΔV (V_2_–V_1_ in V), and the scan rate ν (in V s^−1^).

(2) $$C_{s}=\frac{\int_{V_{1}}^{V_{2}}I\left(V\right)dV}{m\Delta V\nu }$$

The specific capacitance of the measured electrodes based on GCD measurements was calculated from the discharge area using Equation (3), including the applied current I (in A), the discharge area, the mass m, and the potential or voltage window ΔV (V_2_–V_1_ in V).

(3) $$C_{s}=\frac{2I\int_{t_{1}}^{t_{2}}V\left(t\right)dt}{m\Delta V^{2}}$$

## Conflict of Interest

The authors declare no conflict of interest.

## Author Contributions

N.O. and M.B.C. contributed equally to this work. A.F. initiated this research, developed the idea, acquired third‐party funding, and supervised the team. A.F., N.O., M.B.C., O.B., and E.S.B. designed the experiments. N.O. synthesized the (meso)porous *N*‐doped carbon nanospheres and performed material characterization. M.B.C. and N.A. prepared electrodes and performed electrochemical measurements. O.B. prepared cross sections and took SEM images. S.E.B. conducted high‐resolution TEM measurements. A.F., N.O., and M.C.B. designed the manuscript storyline. N.O. wrote the manuscript with input from all authors. All authors discussed and revised the manuscript and have given approval to its final version.

## Supporting information



Supporting Information

## Data Availability

The data that support the findings of this study are available from the corresponding author upon reasonable request.
