# Supplementary material for: Pore Size Independent Particle Size Control of Mesoporous N‐doped Carbon Nanospheres for 3D Bottom‐Up Electrode Design
Source: Small. 2025 Nov 29;22(1):e06253. doi: 10.1002/smll.202506253 (PMC12757992; doi:10.1002/smll.202506253)
Supplement: Supplementary file 1 — Supporting Information [file SMLL-22-e06253-s001.docx]

Pore Size Independent Particle Size Control of Mesoporous N-doped Carbon Nanospheres for 3D Bottom-Up Electrode Design

Niklas Ortlieb^#^, María B. Camarada^#^, Olivia Basu, Hafiz M. N. Amin, S. Esmael Balaghi
and Anna Fischer*

N. Ortlieb, M.B. Camarada, O. Basu, S.E. Balaghi, H.M.N. Amin, A. Fischer

Institute of Inorganic and Analytic Chemistry, University of Freiburg, Albertstraße 21, 79104 Freiburg, Germany

E-mail: anna.fischer@ac.uni-freiburg.de

N. Ortlieb, M.B. Camarada, O. Basu, S.E. Balaghi, H.M.N. Amin, A. Fischer

Freiburg Center of Interactive Materials and Bioinspired Technologies (FIT), University of Freiburg, Georges-Köhler-Allee 105, 79110 Freiburg, Germany

N. Ortlieb, M.B. Camarada, O. Basu, S.E. Balaghi, H.M.N.Amin, A. Fischer

Cluster of Excellence livMatS, University of Freiburg, Georges-Köhler-Allee 105, 79110 Freiburg, Germany

N. Ortlieb, S.E. Balaghi, A. Fischer

Freiburg Materials Research Center (FMF), University of Freiburg, Stefan-Meier-Str. 21, 79104

^#^ both authors contributed equally to this work

Keywords: mesoporous N-doped carbon nanospheres, particle size control, 3D bottom-up electrode design, electrochemical energy conversion and storage, supercapacitors


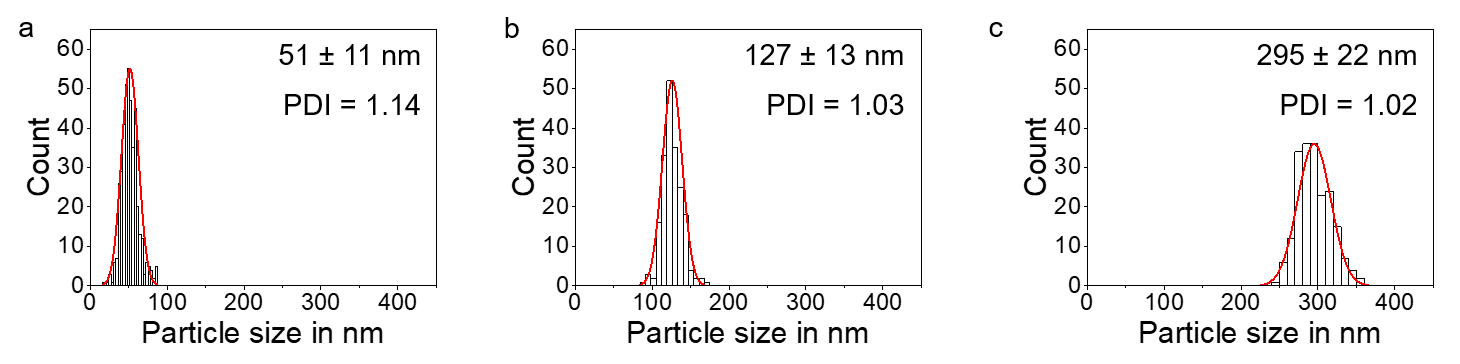
**Figure S1:** Particle size distributions of the synthesized MPNC nanospheres with different particle sizes. MPNC-7-50-1000 (a), MPNC-7-130-1000 (b) and MPNC-7-300-1000 (c).


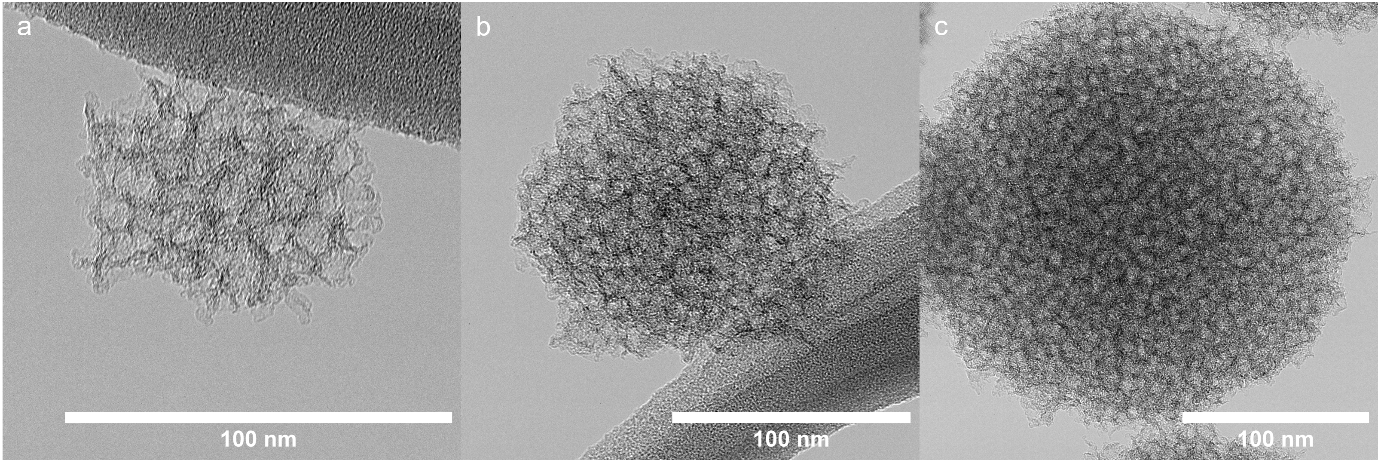
**Figure S2:** HRTEM images of the synthesized MPNC nanospheres with different particle sizes. MPNC-7-50-1000 (a), MPNC-7-130-1000 (b) and MPNC-7-300-1000 (c).


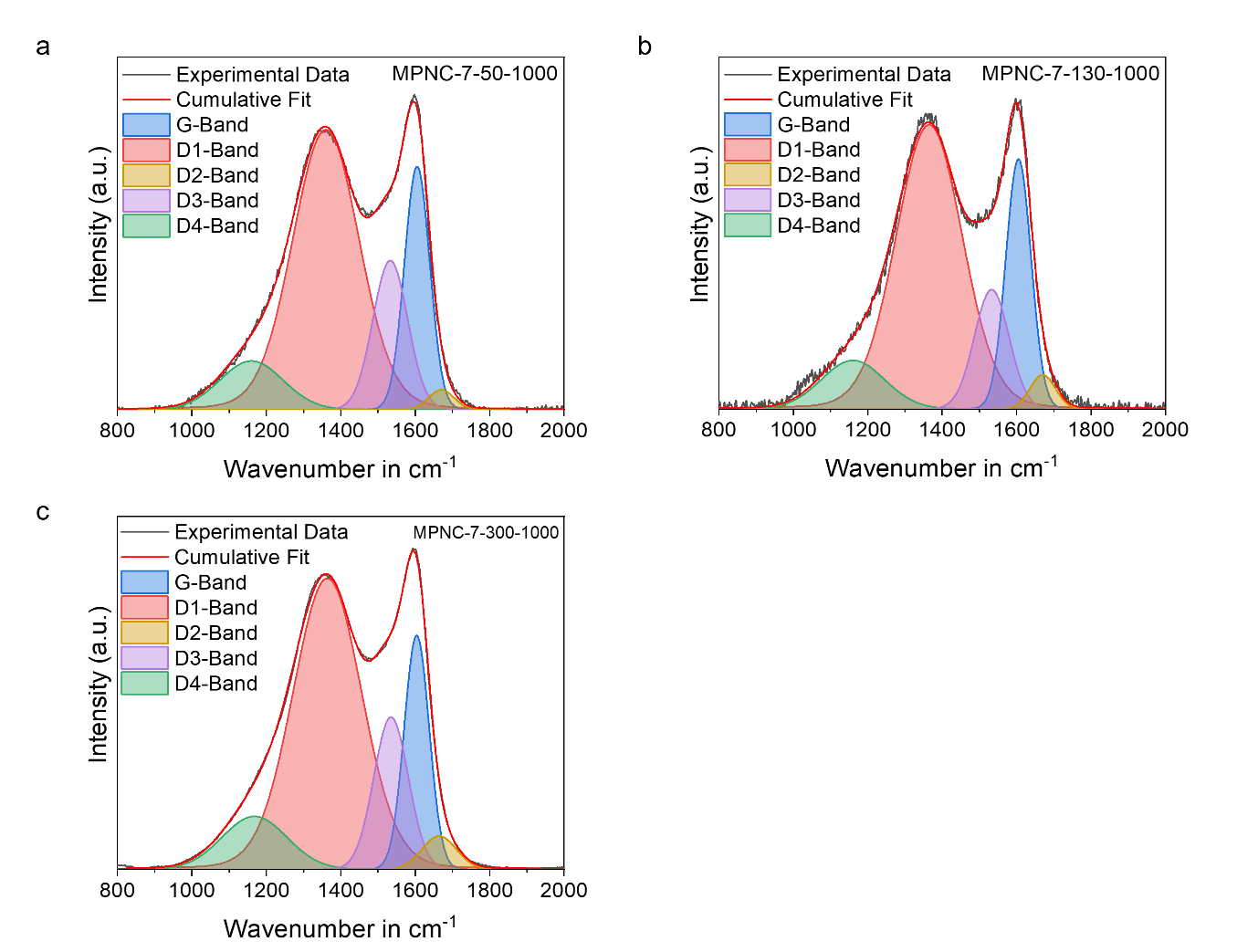
**Figure S3:** Deconvoluted Raman spectra of the MPNC nanospheres with different particle sizes for MPNC-7-50-1000 (a), MPNC-5-130-1000 (b) and MPNC-7-300-1000 (c). The obtained Raman spectra need to be fitted with a five-band model.


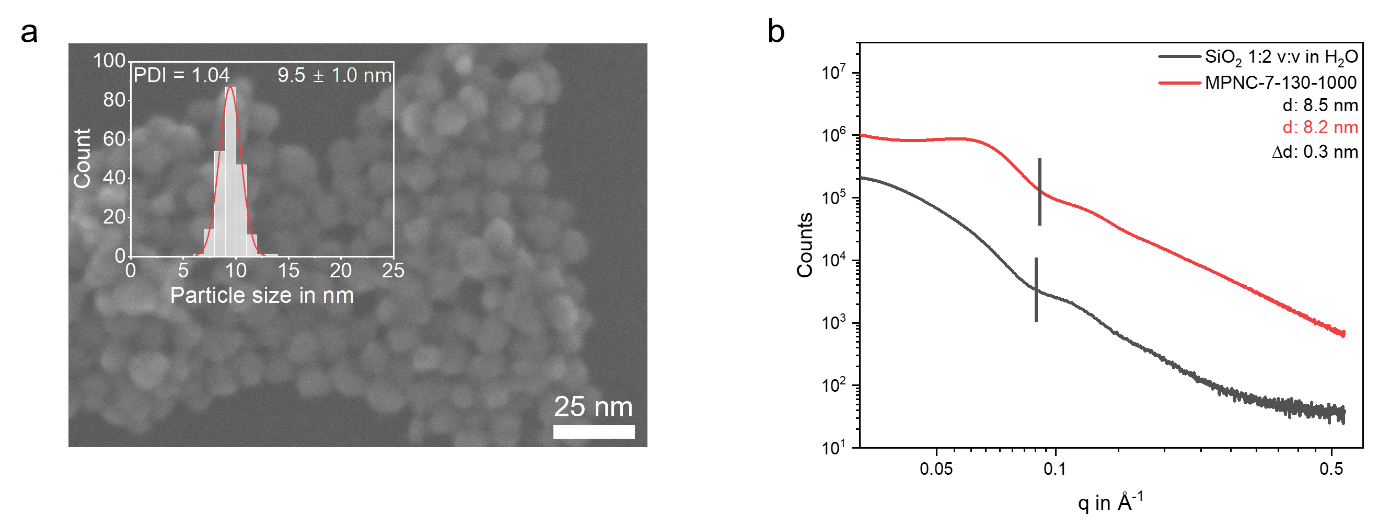
**Figure S4:** Particle size determination of the used SiO_2_ template using SEM (a) and SAXS (b) measurements.

**Table S1:** Proportions of the deconvoluted Raman bands to the total integral of the Raman spectrum for the MPNC nanospheres with different particle sizes.

|  | **D1 band %** | **D2 band %** | **D3 band %** | **D4 band %** | **G band %** |
| --- | --- | --- | --- | --- | --- |
| MPNC-7-50-1000 | 56.4 | 1.5 | 15.1 | 9.0 | 18.0 |
| MPNC-7-130-1000 | 55.8 | 1.9 | 14.4 | 11.0 | 16.9 |
| MPNC-7-300-1000 | 57.6 | 1.9 | 14.0 | 9.2 | 17.3 |

**Table S2:** Elemental composition determined by elemental combustion analysis for the MPNC nanospheres with different particle sizes.

|  | **C wt-%** | **H**  **wt-%** | **N**  **wt-%** | **S**  **wt-%** |
| --- | --- | --- | --- | --- |
| MPNC-7-50-1000 | 80.2 | 1.5 | 4.7 | 0.4 |
| MPNC-7-130-1000 | 83.0 | 1.6 | 5.3 | 0.7 |
| MPNC-7-300-1000 | 81.9 | 1.4 | 4.7 | 0.5 |

**Table S3:** Nitrogen physisorption data of the MPNC nanospheres with different particle sizes.

|  | **Total surface area (BET)**  **m^2^g^-1^** | **Total surface area (DFT)**  **m^2^g^-1^** | **Surface area (micropore fraction) (DFT) m^2^g^-1^** | **Surface area (mesopore fraction) (DFT) cm^3^g^-1^** | **Total pore volume**  **(BET) cm^3^g^-1^** | **Total pore volume**  **(DFT) cm^3^g^-1^** | **Pore volume (micropore fraction) (DFT) cm^3^ g^-1^** | **Pore volume (mesopore fraction DFT) cm^3^g^-1^** |
| --- | --- | --- | --- | --- | --- | --- | --- | --- |
| MPNC-7-50-1000 | 965 | 1028 | 406 | 622 | 2.439 | 1.903 | 0.149 | 1.754 |
| MPNC-7-130-1000 | 967 | 963 | 413 | 550 | 1.481 | 1.230 | 0.157 | 1.073 |
| MPNC-7-300-1000 | 945 | 921 | 320 | 601 | 1.341 | 1.213 | 0.127 | 1.086 |


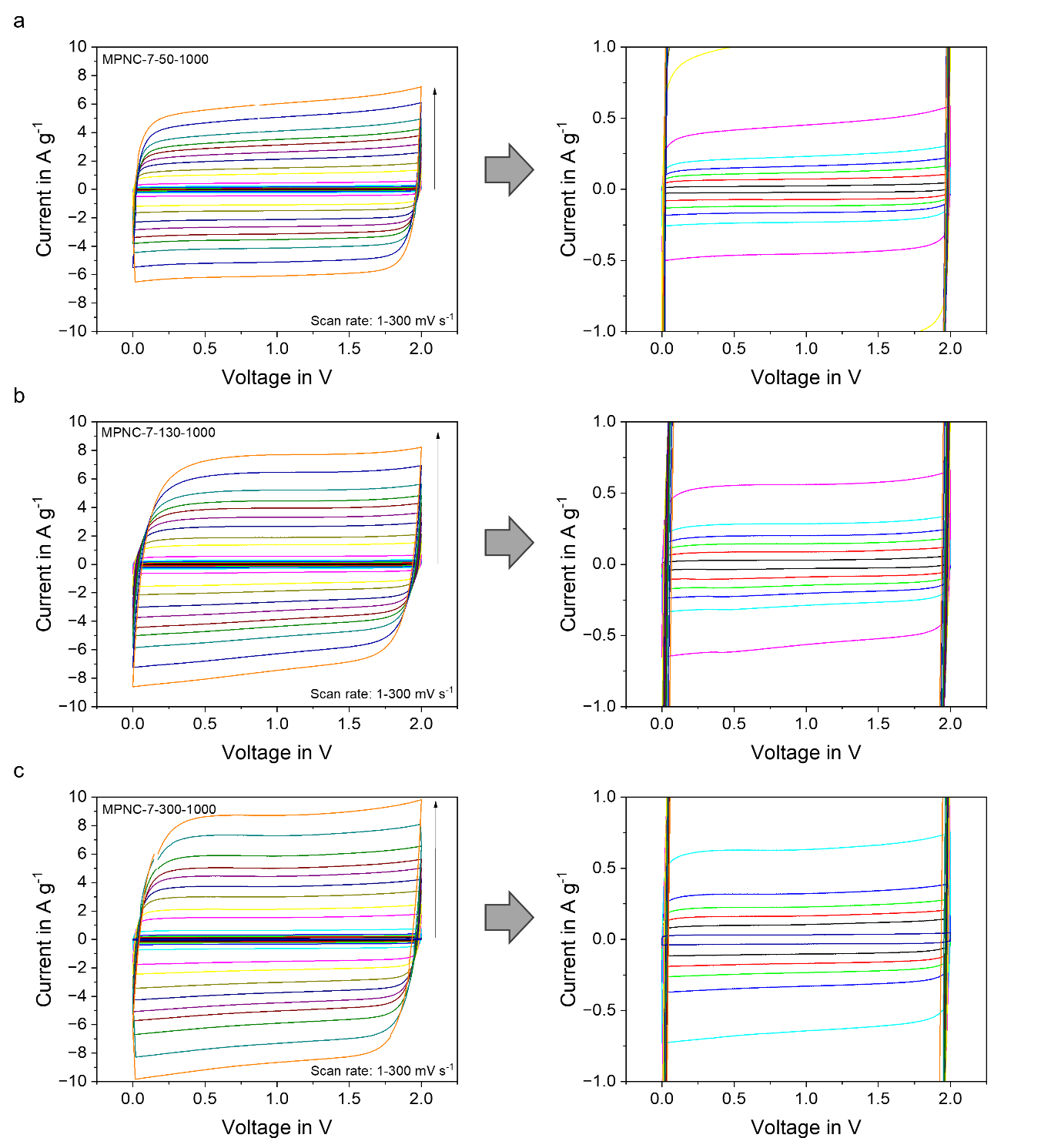
**Figure S5:** Evaluation of the performance of the MPNC-based EDLCs in the coin cell housings using 1.0 M LiPF6 EC/DEC electrolyte. Cyclic voltammetry curves for the MPNC nanospheres with different particle sizes at different scan rates between 1-300 mV s^-1^ for electrodes based on MPNC-7-500-1000 (a), MPNC-7-130-1000 (b) and MPNC-7-300-1000 (c).


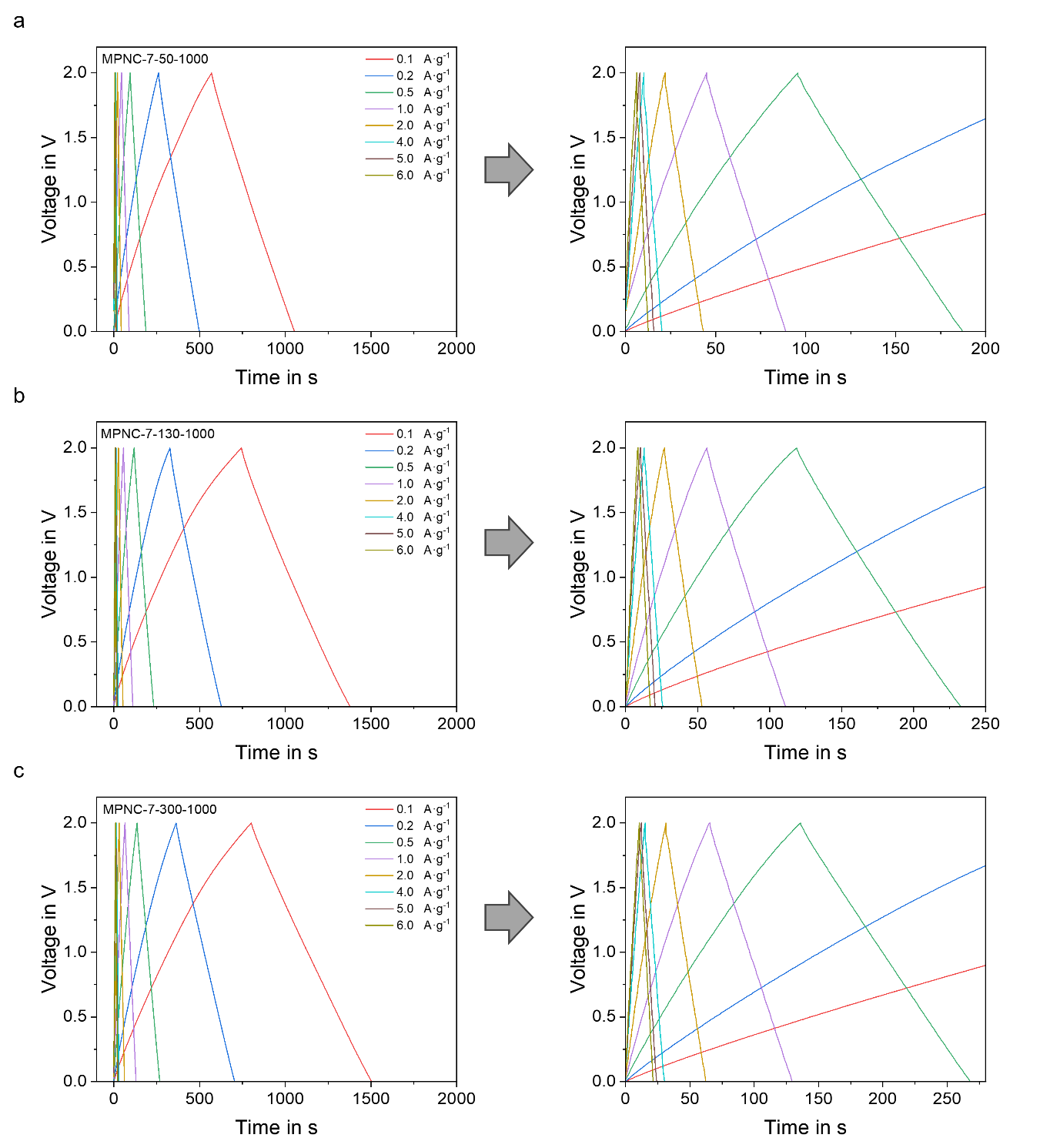
**Figure S6:** Evaluation of the rate capability of the MPNC-based EDLCs in coin cell housings using 1.0 M LiPF6 EC/DEC electrolyte. Charge/discharge time of the MPNC nanospheres with different pore sizes at different charge/discharge currents between 0.1 and 6.0 A g^-1^ for electrodes based on MPNC-7-500-1000 (a), MPNC-7-130-1000 (b) and MPNC-7-300-1000 (c).

**Table S4:** EDLC performance of the MPNCs nanospheres with different particle sizes and a constant pore size around 9 nm in two electrode coin cells. 1.0 M LiPF6 in EC/DEC was used as electrolyte. CV and GCD measurements were performed in the potential window between 0 and 2 V.

| **Sample name** | **Physisorption** | **Cyclic Voltammetry** | | **Galvanostatic Charge Discharge** | | |
| --- | --- | --- | --- | --- | --- | --- |
|  | **Specific surface area  m^2^ g^-1^** | **Scan rate   mV s^-1^** | **Specific capacitance   F g^-1^** | **Specific capacitance at 0.1 A g^-1^  F g^-1^** | **Specific capacitance at 8 A g^-1^  F g^-1^** | **Specific rate capacitance retention at 8 A g^-1^ %** |
| MPNC-7-50-1000 | 965 | 1 | 48 | 48 | 37 | 77 |
| MPNC-7-130-1000 | 967 | 1 | 61 | 57 | 47 | 83 |
| MPNC-7-300-1000 | 945 | 1 | 69 | 67 | 56 | 84 |
